# Metabolic Remodeling of the Tricarboxylic Acid Cycle and Glycolysis Reveals Cold-Induced Respiratory Adaptations in *Streltzoviella insularis* (Staudinger) (Lepidoptera: Cossidae) Larvae

**DOI:** 10.3390/insects16080864

**Published:** 2025-08-19

**Authors:** Lingxu Zhi, Ruixin Li, Baosheng Zhang, Yan Zhang, Jiahe Pei, Shixiang Zong

**Affiliations:** 1 Beijing Key Laboratory for Forest Pest Control, School of Forestry, Beijing Forestry University, Beijing 100083, China; zhilingxu1685@163.com (L.Z.); 13146891722@163.com (R.L.); 2 Jungar Banner Forestry and Grassland Development Center, Jungar 017299, China; zongsx@126.com (B.Z.); wu624651232@163.com (Y.Z.)

**Keywords:** *Streltzoviella insularis*, overwintering, rate-limiting enzymes, metabolites, anaerobic respiration

## Abstract

Global climate change is pushing insects into colder regions. Understanding their cold tolerance is important for predicting population dynamics. During overwintering, *Streltzoviella insularis* larvae activate the AMPK signaling pathway. This suggests that energy metabolism plays a key role under cold stress. In this study, we used enzyme activity assays, LC-MS-based targeted metabolomics, and transcriptome sequencing. We focused on six key enzymes in glycolysis and the TCA cycle. We also measured related metabolites and regulatory genes. Hexokinase (HK) and citrate synthase (CS) activities were highly sensitive to temperature. HK increased then markedly decreased; CS was significantly downregulated. Pyruvate kinase (PK), isocitrate dehydrogenase (IDH), and α-ketoglutarate dehydrogenase (KGD) showed trends that matched changes in larval cold tolerance, exhibiting an up–down–up expression trend. Glycolytic metabolites (glucose-6-phosphate, fructose-6-phosphate, 1,6-fructose-diphosphate, phosphoenolpyruvic acid) peaked at −10 °C. TCA intermediates (citrate, acetyl-CoA, α-ketoglutaric acid, and isocitrate) were more abundant at 0–4 °C. Pyruvate increased significantly. PYR content showed a significant increase followed by a decrease, peaking at 0 °C. It was converted into lactate and acetyl-CoA. ATP levels dropped and then increased, reaching their lowest level at 0 °C. These results suggest a shift from aerobic to mixed aerobic–anaerobic metabolism. Transcriptome data showed differential expression of key metabolic genes such as *phosphoenolpyruvate carboxykinase*, *phosphoglycerate kinase*, and *ATP synthase subunit beta*. These gene changes supported the trends in enzymes and metabolites. Our findings reveal a coordinated metabolic and transcriptional response to cold. This provides a basis for understanding the cold adaptation and potential range expansion of *S. insularis*.

## Simple Summary

Global warming drives insect expansion into colder regions, highlighting the need to understand cold tolerance. *Streltzoviella insularis* larvae activate the AMPK pathway during overwintering, suggesting key metabolic regulation. This study used enzyme activity assays, LC-MS metabolomics, and transcriptomics to examine changes in glycolysis and the tricarboxylic acid cycle. Hexokinase, citrate synthase, pyruvate kinase, isocitrate dehydrogenase, and α-ketoglutarate dehydrogenase were temperature-sensitive enzymes. Hexokinase activity increased and then markedly decreased, while citrate synthase activity was significantly downregulated. The activities of the other three enzymes, all associated with cold tolerance, exhibited an up–down–up expression trend. Glycolytic metabolites peaked at −10 °C, and TCA metabolites peaked at 0 °C–4 °C. Pyruvate accumulation, conversion to lactate and acetyl-CoA, and ATP fluctuation suggest a shift to facultative anaerobic metabolism. Differential expression of *phosphoenolpyruvate carboxykinase*, *phosphoglycerate kinase*, and *ATP synthase subunit beta* supported these changes. Overall, we reveal coordinated metabolic and transcriptional adjustments supporting cold adaptation in *S. insularis* larvae.

## 1. Introduction

Climate change intensifies the variability of pest outbreaks, complicating the prediction and management of insect population dynamics. These environmental changes impact pest populations by altering key biological traits, including life history traits, physiological processes, reproductive capacities, and interactions with host trees and natural enemies. Temperature fluctuations play a pivotal role in this process, as insects exhibit high physiological sensitivity to temperature fluctuations, which directly affects their survival rates [1,2,3]. An insect’s tolerance to low-temperature stress depends on its ability to resist cold-induced injury at critical temperature thresholds. [4]. Cold hardiness, defined as an organism’s ability to endure prolonged or brief exposure to low temperatures, is a critical determinant of insect survival in cold environments [5]. Insects have evolved distinct cold-tolerance strategies, broadly categorized as freeze-tolerant, freeze-avoidant, and chill-susceptible [6,7]. As ectotherms, insects in temperate and polar regions must employ overwintering strategies to survive habitat-specific cold extremes. Recent climatic shifts have led to an increased frequency of extreme temperature events (e.g., anomalously warm summers and severe winters) [8,9], causing species populations to either expand or contract in response to temperature variations [10,11].

*Streltzoviella insularis* is a wood-boring pest that infests a range of tree species, including *Fraxinus pennsylvanica*, *Sophora japonica*, and *Ginkgo biloba*, as well as other trees widely used in urban greening and afforestation projects across northern China. This species is distributed across multiple regions, notably Beijing, Shanghai, Hebei, and Shandong [12]. Its range has expanded recently, with its first documented occurrence in Xinjiang in 2023 [13], and evidence suggests that it may continue to spread into colder regions. The larvae overwinter within tree trunks, where they aggregate and feed on both the trunk and branches of host trees, potentially leading to tree mortality under severe infestation conditions.

Previous research has identified *S. insularis* as a freeze-tolerant species, with a supercooling point of approximately −5 °C [14]. During overwintering, the AMPK signaling pathway is activated in larvae, promoting increased catabolic activity. Cellular respiration, a fundamental component of insect catabolism, plays a central role in cold adaptation, with the tricarboxylic acid (TCA) cycle recognized as a key metabolic pathway involved in cold tolerance in this species [14,15]. Numerous studies have underscored the critical role of metabolic regulation in insect cold hardiness. For example, in species such as *Corythucha ciliata* and *Thaumatotibia leucotreta*, cold adaptation has been associated with the activation of the AMPK pathway and upregulation of glycolysis, gluconeogenesis, and the TCA cycle. Moreover, hypoxic conditions have been shown to amplify the anaerobic metabolic responses induced by low temperatures [16,17].

Although wood-boring pests benefit from protective shelters and abundant food resources during winter, studies on their respiratory energy metabolism remain limited. This study bridges this knowledge gap by systematically examining the role of cellular respiration in cold hardiness through a comprehensive analysis of key rate-limiting enzymes in larval energy metabolism. Specifically, we examined hexokinase (HK), phosphofructokinase (PFK), and pyruvate kinase (PK) in the glycolytic pathway, as well as citrate synthase (CS), isocitrate dehydrogenase (IDH), and α-ketoglutarate dehydrogenase (KGD) in the TCA cycle, along with their associated metabolites. In addition, transcriptomic sequencing was performed to identify differentially expressed genes related to energy metabolism, providing further insight into the molecular mechanisms of cold adaptation. This research addresses this gap by elucidating metabolic adaptations that support overwintering survival. Furthermore, these findings provide a theoretical framework for identifying upstream regulatory targets, contributing to developing novel and more effective pest control strategies.

## 2. Materials and Methods

### 2.1. Insect Collection

On 1 April 2023, *Fraxinus pennsylvanica* logs infested with *S. insularis* larvae were collected from Wutuan Village, Tongzhou District, Beijing (39°32′ N, 116°32′ E). Following log splitting, larvae were reared in an artificial incubator with a synthetic diet. The composition of the diet is detailed in Table S1 [18].

The rearing temperature was maintained at a constant 25 °C under complete darkness to promote the development of healthy larvae.

### 2.2. Low-Temperature Treatment

After 122 days of rearing, size- and weight-matched larvae (body length: 3.7–4.5 cm; body weight: 0.7–1.1 g) were subjected to low-temperature treatments. All individuals were pre-acclimated at 4 °C for 7 days to avoid direct cold shock. Larvae were then randomly assigned to four treatment groups (*n* = 60 per group) and exposed to constant temperatures of 4 °C, 0 °C, −5 °C, or −10 °C for 7 days. Each group was maintained in climate-controlled metal boxes (22.8 cm × 15.3 cm × 5.9 cm) containing a synthetic diet.

The 4 °C condition served as the experimental control based on two criteria: (1) it reflects typical overwintering temperatures (0–5 °C) for temperate insects [19], and (2) it results in minimal cold injury in *S. insularis* larvae [14]. The mortality rate under temperature treatment remains unclear. This study focuses on the transient accumulation of substances and gene changes following treatment, and since the insects did not recover to room temperature, their mortality rate could not be determined. Further research will be conducted to clarify these aspects in greater detail.

Following the temperature treatments, all surviving larvae were immediately transferred to upright-positioned centrifuge tubes, flash-frozen in liquid nitrogen, and stored at either −40 °C for subsequent enzyme activity assays or −80 °C for metabolite quantification.

### 2.3. Enzyme Activity Assay in Larvae at Low Temperature

Enzyme activity assays were performed on *S. insularis* larvae from each temperature group (Table 1). Due to the limited tissue available per larva and the need to analyze multiple enzymes, three individuals per group were used as biological replicates, each with three technical replicates. CS and HK activities were measured by Shanghai Meiji Bio-pharmaceutical Technology Co., Ltd. (Shanghai, China) using commercial assay kits. Citrate synthase (CS) activity was determined with a double-antibody sandwich ELISA kit (YT-927855O2/48T, Jiangsu Yutong Biotechnology Co., Ltd., Changzhou, China). Hexokinase (HK) activity was assessed using an ELISA kit specifically for HK. α-Ketoglutarate dehydrogenase (KGD) and phosphofructokinase (PFK) activities were measured using commercial kits (BC0710-50T/48S and BC0530-50T/48S, respectively; Beijing Solarbio Technology Co., Ltd., Beijing, China). Pyruvate kinase (PK) activity was measured with kit AC10162-50T/48S (Shanghai Jizi Biochemical Technology Co., Ltd., Shanghai, China), and isocitrate dehydrogenase (IDH) activity with kit AK328-50T/48S (Beijing Boosun Biotechnology Co., Ltd., Beijing, China). Detailed procedures are provided in Supplementary Materials (Enzyme Assay Steps 1–6).

### 2.4. Changes in Energy Metabolites Under Low-Temperature Conditions

#### 2.4.1. Determination of Glucose Content

Glucose content was measured in *S. insularis* larvae from the 4 °C, 0 °C, −5 °C, and −10 °C treatment groups. Approximately 0.1 g of tissue from each larva was homogenized in 0.9 mL of PBS on ice. Five individuals per group were used as biological replicates, each with three technical replicates. Glucose levels were determined using a commercial assay kit (R30282-100T, Shanghai Yuanye Biotechnology Co., Ltd., Shanghai, China). Detailed procedures are provided in the Supplementary Materials (Glucose Assay Step 7).

#### 2.4.2. Determination of the Metabolites

Metabolomic analysis requires minimal tissue per sample, enabling larger sample sizes. Five *S. insularis* larvae were used as biological replicates for each temperature treatment. Key metabolites from the TCA cycle and glycolysis were quantified using targeted LC-MS-based metabolomics. A total of 18 metabolites were included (Table 2).

Samples (60 ± 5 mg) were taken from −80 °C storage, placed in homogenization tubes with pre-chilled methanol (200 μL), 10 mM succinic acid-D6 internal standard (10 μL, Sigma-Aldrich, St. Louis, MO, USA), and two 4 mm black ceramic beads. After quenching with liquid nitrogen (5 s), samples were homogenized three times (20 s each), followed by the addition of cold chloroform (400 μL), vortexing (30 s), sonication in ice (5 min), and another vortex (10 min). Deionized water (100 μL) was added, followed by vortexing (10 min). After centrifugation (14,000× *g*, 10 °C, 20 min), 200 μL of supernatant was collected, dried under vacuum or nitrogen, and stored at −80 °C. For analysis, samples were reconstituted in 100 μL of acetonitrile/water (1:1), vortexed (30 s), centrifuged (14,000× *g*, 10 °C, 15 min), and the supernatant was analyzed. An ACQUITY UPLC BEH Amide column (1.7 µm, 2.1 × 150 mm; Waters, Wexford, Ireland) was used with a 1290 Infinity UPLC system (Agilent, Santa Clara, CA, USA) coupled to a 6500/5500 QTRAP mass spectrometer (SCIEX). LC conditions: autosampler at 4 °C, column at 35 °C, mobile phase A: 50 mM ammonium acetate with 1.2% ammonium hydroxide; B: acetonitrile with 1% acetylacetone; flow rate: 300 μL/min; injection volume: 2 μL. MS settings: source temp 450 °C, Gas 1/2: 45, Curtain Gas: 30, ISVF: −4500 V. Detection was performed in MRM mode. All sample preparations and analyses were conducted by Panomix Biomedical Technology (Suzhou, China).

Metabolite concentrations were determined using an isotope-labeled internal standard method. The absolute content of each metabolite was calculated by normalizing the ratio of the target compound’s peak area to that of the internal standard, followed by adjustment based on the internal standard concentration. Quantified metabolite extraction and analysis were performed using MultiQuant 3.0.2 software, which enabled the identification of chromatographic peak areas and retention times.

### 2.5. Transcriptome Sequencing

RNA-seq data were processed for quality control, alignment, and transcript quantification. *S. insularis* larvae from the 4 °C, 0 °C, −5 °C, and −10 °C treatments were selected for RNA-seq, with three biological replicates per group. Total RNA was extracted using the Aidlab Tissue and Cell RNA Rapid Extraction Kit (Cat. No. RN28, Aidlab Beijing China). RNA concentration and purity were assessed using a NanoDrop 2000 (Thermo Fisher Scientific, Waltham, MA, USA), and integrity was evaluated with an Agilent 5300 Bioanalyzer (Santa Clara, CA, USA). Only samples meeting quality criteria (OD260/280 = 1.8–2.2, OD260/230 ≥ 2.0, RQN ≥ 6.5, 28S:18S ≥ 1.0, RNA > 1 μg; or RIN > 6.5, RNA > 10 ng) were used for library preparation.

Libraries were constructed using the Illumina^®^ Stranded mRNA Prep, Ligation kit (San Diego, CA, USA) with 1 μg of total RNA. Poly(A)+ mRNA was enriched with oligo(dT) magnetic beads, fragmented, and reverse-transcribed into double-stranded cDNA. After end repair, phosphorylation, and adapter ligation, 300–400 bp fragments were selected and PCR-amplified (10–15 cycles). Libraries were quantified using Qubit 4.0 and sequenced.

Raw reads were trimmed and quality-filtered with fastp (https://github.com/OpenGene/fastp, accessed on 27 December 2024). Clean reads were assembled de novo using Trinity (https://github.com/trinityrnaseq/trinityrnaseq/wiki, accessed on 27 December 2024), filtered by CD-HIT (http://weizhongli-lab.org/cd-hit/, accessed on 27 December 2024) and TransRate (http://hibberdlab.com/transrate/, accessed on 27 December 2024), and evaluated with BUSCO (Benchmarking Universal Single-Copy Orthologs, http://busco.ezlab.org, accessed on 27 December 2024). Functional annotation was performed using Diamond (https://github.com/bbuchfink/diamond, accessed on 28 December 2024) against NR and KEGG databases (E < 1.0 × 10^−5^), and GO annotation was conducted using HMMER (ftp://selab.janelia.org/pub/software/hmmer3/3.0/hmmer-3.0.tar.gz, accessed on 28 December 2024).

### 2.6. Screening of Differentially Expressed Genes and Identification of Key Genes

Differential expression analysis was performed using DESeq2 by comparing the 0 °C, −5 °C, and −10 °C treatment groups with the 4 °C control group. Genes with |log_2_FC| ≥ 1 and FDR-adjusted *p* < 0.05 were considered significantly differentially expressed.

Differentially expressed genes (DEGs) were further filtered based on their functional enrichment and overlap with pathway-specific gene sets, including glycolysis, the tricarboxylic acid (TCA) cycle, oxidative phosphorylation, and pyruvate metabolism. These gene sets were constructed using the Majorbio platform. A total of nine DEGs were selected for qPCR analysis: *GAPDH*, *LDH1*, *ADH1*, *ADH3*, *ALDH*, *PEPCK*, *ATPB*, *NDUFS3*, and *DLST2*, all of which encode key metabolic enzymes potentially involved in cold adaptation (abbreviations and full gene names are listed in Supplementary Materials Table S2).

### 2.7. Statistical Analysis

All statistical analyses were performed using IBM SPSS Statistics 26.0. Data normality and homogeneity of variance were verified prior to one-way ANOVA followed by LSD post hoc tests (significance levels: * *p* < 0.05). All error bars in the visualized data represent standard deviations (SDs). Metabolic pathway enrichment analysis was conducted using the MetaboAnalyst 5.0 platform (https://www.metaboanalyst.ca/MetaboAnalyst/faces/home.xhtml, accessed on 19 April 2024). Transcriptome data were analyzed via the Majorbio Bioinformatics platform (https://analysis.majorbio.com/drna/specimen_general/task_id/18ro_49gsfss13gdseojfmlclp3, accessed on 21 November 2024). Relationships between enzyme activities and metabolites were modeled using quadratic polynomial regression in the Python 3.9 (64-bit) environment, with direct visualization of results. Final figures were prepared using GraphPad Prism 10.

## 3. Results

### 3.1. Changes in Enzyme Activity in the TCA Cycle and Glycolysis

**Scheme 1:**
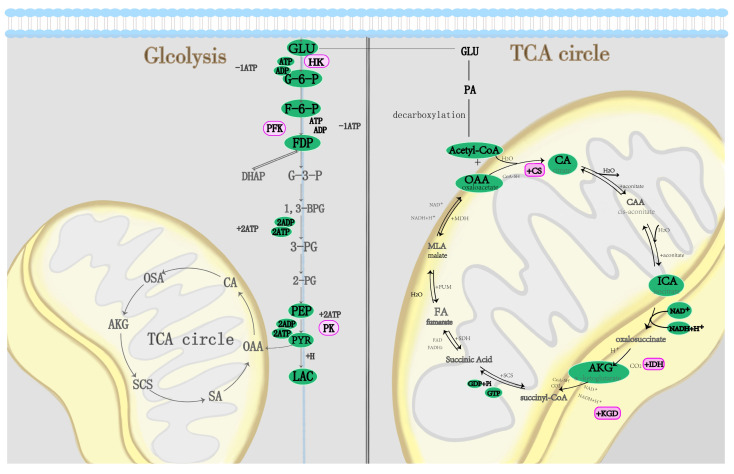
Schematic diagram of the tricarboxylic acid (TCA) cycle and glycolytic metabolic pathways. We selected 18 metabolites associated with those mentioned above key rate-limiting enzymes. Metabolites involved in the TCA cycle and glycolysis are highlighted in green, while rate-limiting enzymes are shown in purple. Abbreviations: GLU, glucose; G-6-P, glucose-6-phosphate; F-6-P, fructose-6-phosphate; FDP, 1,6-fructose-diphosphate; PEP, phosphoenolpyruvic acid; PYR, pyruvic acid; LAC, lactic acid; acetyl-CoA, acetoacetyl-CoA; OAA, oxaloacetic acid; CA, citric acid; ICA, isocitric acid; AKG, α-ketoglutaric acid; ATP, adenosine triphosphate; ADP, adenosine diphosphate; GTP, guanosine triphosphate; GDP, guanosine diphosphate; NADH, reduced nicotinamide adenine dinucleotide; NAD^+^, oxidized nicotinamide adenine dinucleotide.

The specific locations of the detected enzymes and metabolites are shown in Scheme 1.

**Figure 1:**
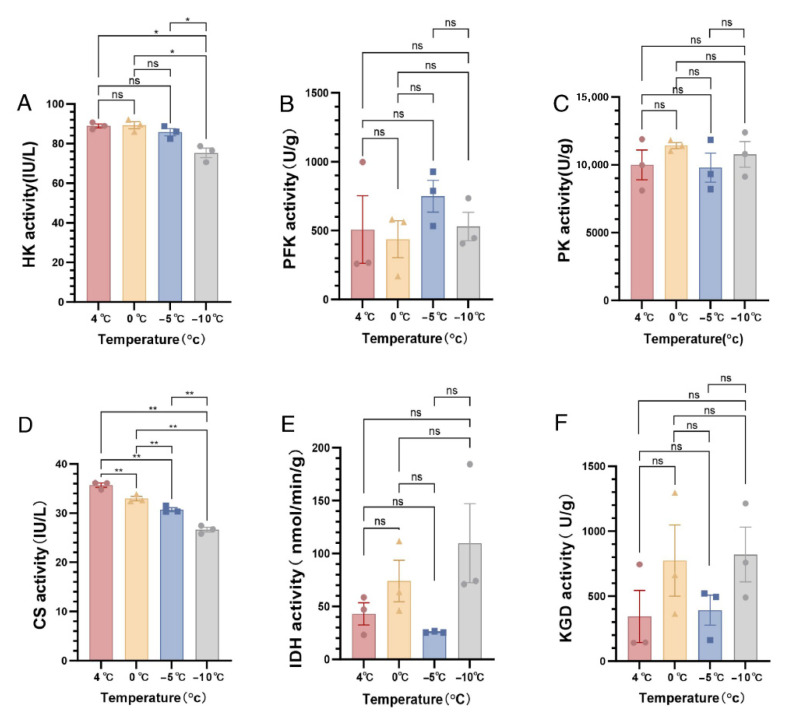
Trends in the activity of rate-limiting enzymes measured at four temperatures (4 °C, 0 °C, −5 °C, and −10 °C). All data points presented in the figures represent biological replicates, and each bar in the bar charts reflects the mean value of the corresponding biological replicates within each treatment group. Error bars indicate standard deviations. Statistical significance was determined by one-way ANOVA followed by the LSD test. Asterisks indicate significant differences compared with the 4 °C group (* *p* < 0.05; ** *p* < 0.01, ns > 0.05). (**A**) Hexokinase (HK) activity. (**B**) Phosphofructokinase (PFK) activity. (**C**) Pyruvate kinase (PK) activity. (**D**) Citrate synthase (CS) activity. (**E**) Isocitrate dehydrogenase (IDH) activity. (**F**) α-Ketoglutarate dehydrogenase (KGD) activity.

The activity of HK significantly increased as the temperature decreased from 4 °C to 0 °C, reaching a peak at 0 °C, and then significantly declined with a further temperature decrease, reaching the lowest level at −10 °C (Figure 1A) (F_3,8_ = 12.866, *p* < 0.05). CS activity significantly decreased from its highest level at 4 °C to the lowest at −10 °C (Figure 1D) (F_3,8_ = 75.487, *p* < 0.01). In contrast, the activities of PFK, PK, IDH, and KGD did not show statistically significant differences among the temperature treatments (PFK: F_3,8_ = 0.730, *p* = 0.563; PK: F_3,8_ = 0.676, *p* = 0.591; IDH: F_3,8_ = 2.898, *p* = 0.102; KGD: F_3,8_ = 1.440, *p* = 0.302) (Figure 1B,C,E,F). The activities of HK and CS were significantly affected by decreasing temperatures. The activities of PK, IDH, and KGD exhibited a fluctuating trend of increase, decrease, and subsequent increase, which was consistent with the pattern of cold tolerance observed in *Streltzoviella insularis* larvae under different temperature treatments.

### 3.2. Changes in Metabolite Concentration

**Figure 2:**
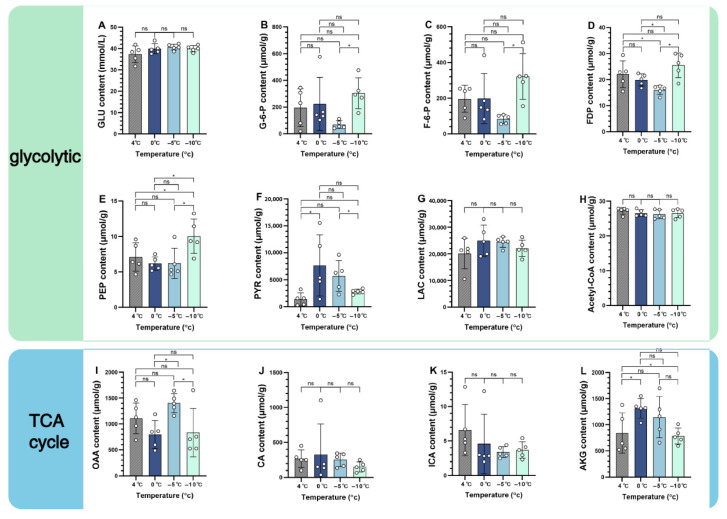
Changes in glycolysis- and tricarboxylic acid (TCA) cycle-related metabolite levels under different temperature conditions. Metabolite concentrations were measured at four temperatures (4 °C, 0 °C, −5 °C, and −10 °C). Each bar represents the mean of five biological replicates (*n* = 5), with each replicate calculated from three technical repeats. Error bars indicate standard deviations. Statistical significance was determined using one-way ANOVA followed by the LSD post hoc test. Significant differences between groups are indicated by asterisks (*), while “ns” denotes non-significant differences (*p* < 0.05). (**A**) Glucose (GLU). (**B**) Glucose-6-phosphate (G-6-P). (**C**) Fructose-6-phosphate (F-6-P). (**D**) Fructose-1,6-diphosphate (FDP). (**E**) Phosphoenolpyruvic acid (PEP). (**F**) Pyruvic acid (PYR). (**G**) Lactic acid (LAC). (**H**) Acetyl coenzyme A (acetyl-CoA). (**I**) Oxaloacetic acid (OAA). (**J**) Citric acid (CA). (**K**) Isocitric acid (ICA). (**L**) α-Ketoglutaric acid (AKG).

The contents of F-6-P (Figure 2C; F_3,16_ = 4.471, *p* < 0.05), FDP (Figure 2D; F_3,16_ = 5.596, *p* < 0.05), PEP (Figure 2E; F_3,16_ = 4.274, *p* < 0.05), and PYR (Figure 2F; F_3,16_ = 3.776, *p* < 0.05) showed significant fluctuations in response to decreasing temperatures.

F-6-P and FDP both significantly declined from 4 °C to −5 °C, reaching minimum levels at −5 °C, followed by a significant increase to their peaks at −10 °C; the decrease in FDP from 4 °C to −5 °C was statistically significant.

PEP decreased gradually from 4 °C to a minimum at 0 °C, then significantly increased to its highest level at −10 °C.

PYR increased from 4 °C to a peak at 0 °C and then declined through −5 °C to −10 °C, with overall variation remaining statistically significant.

In contrast, GLU (Figure 2A; F_3_,_16_ = 1.690, *p* = 0.209), G-6-P (Figure 2B; F_3_,_16_ = 2.577, *p* = 0.09), and LAC (Figure 2G; F_3_,_16_ = 1.254, *p* = 0.323) showed no statistically significant changes. However, LAC, derived from PYR under anaerobic conditions, exhibited a similar trend—increasing from 4 °C to 0 °C and then decreasing toward −10 °C.

Acetyl-CoA condenses with oxaloacetate (OAA) (Figure 2I; F_3,16_ = 3.802, *p* < 0.05) to form citrate (CA) (Figure 2J; F_3,16_ = 0.472, *p* = 0.706), thereby initiating the TCA cycle.

The concentration of OAA decreased from 4 °C to 0 °C, reaching its minimum at 0 °C, then significantly increased to a peak at −5 °C, before markedly declining from −5 °C to −10 °C.

The concentration of α-ketoglutarate (AKG) (Figure 2L; F_3,16_ = 3.502, *p* < 0.05) significantly increased from 4 °C to 0 °C, reaching a peak at 0 °C, and then gradually declined to its lowest level at −10 °C.

Acetyl-CoA (Figure 2H; F_3,16_ = 0.683, *p* = 0.576), isocitrate (ICA) (Figure 2K; F_3,16_ = 1.201, *p* = 0.341), and citrate (CA) (Figure 2J; F_3,16_ = 0.472, *p* = 0.706) did not show significant changes. But the metabolites CA, acetyl-CoA, AKG, and ICA exhibited peak contents at 4 °C and 0 °C.

### 3.3. The Relationship Between Rate-Limiting Enzymes and Their Related Metabolites Under Different Temperature Conditions

**Figure 3:**
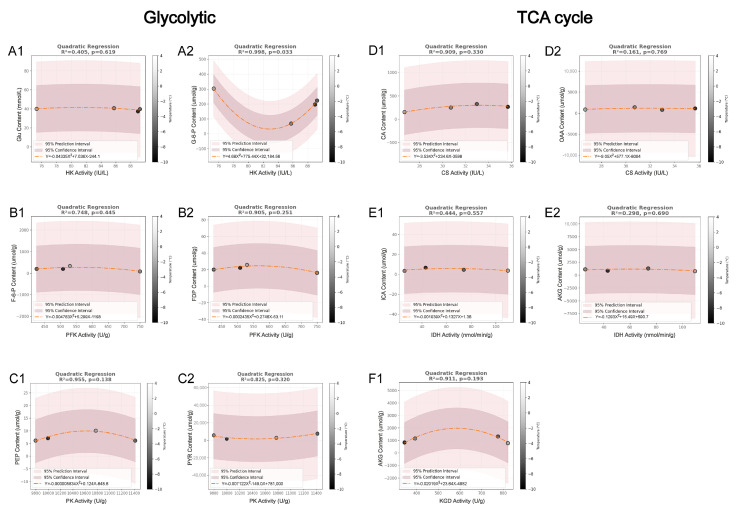
Quadratic regression analysis between enzyme activities and metabolite concentrations in glycolysis and the tricarboxylic acid (TCA) cycle under different temperature conditions. Each subplot displays the relationship between enzyme activity (x-axis) and metabolite concentration (y-axis), fitted with a quadratic regression curve (orange dashed line) and accompanied by 95% confidence and prediction intervals (shaded areas). R2 and *p*-values for each regression are provided. (**A1**): Quadratic regression curves of GIU and HK. (**A2**): Quadratic regression curves of G-6-P and HK. (**B1**): Quadratic regression curves of F-6-P and PFK. (**B2**): Quadratic regression curves of FDP and PFK. (**C1**): Quadratic regression curves of PEP and PK. (**C2**): Quadratic regression curves of PYR and PK. (**D1**): Quadratic regression curves of CA and CS. (**D2**): Quadratic regression curves of OAA and CS. (**E1**): Quadratic regression curves of ICA and IDH. (**E2**): Quadratic regression curves of AKG and IDH. (**F1**): Quadratic regression curves of AKG and KGD.

During glycolysis, HK activity shows a strong quadratic relationship with G6P content (Figure 3A2, R^2^ = 0.998, *p* < 0.05). As HK activity increases, G6P content first decreases and then rises. In contrast, its relationship with GLU content is weaker (Figure 3A1, R^2^ = 0.405, *p* = 0.619), showing a slight increase followed by a decrease.

PFK activity follows a quadratic trend with both F6P (Figure 3B1, R^2^ = 0.748, *p* = 0.445) and FDP (Figure 3B2, R^2^ = 0.905, *p* = 0.251), both increasing and then decreasing. A similar pattern is observed for PK activity and PEP (Figure 3C1, R^2^ = 0.955, *p* = 0.138). However, PK activity and PYR (Figure 3C2, R^2^ = 0.825, *p* = 0.320) show the opposite trend, first decreasing and then increasing. Despite good curve fitting (high R^2^), these relationships lack statistical significance (high *p*-values).

In the TCA cycle, CS activity has a weak quadratic relationship with OAA (Figure 3D2, R^2^ = 0.161, *p* = 0.769). IDH activity shows a mild quadratic trend with ICA (Figure 3E1, R^2^ = 0.444, *p* = 0.557) and AKG (Figure 3E2, R^2^ = 0.298, *p* = 0.690), both rising before declining. In contrast, CS activity correlates more strongly with CA (Figure 3D1, R^2^ = 0.909, *p* = 0.330), and KGD activity follows a similar pattern with AKG (Figure 3F1, R^2^ = 0.911, *p* = 0.193). While these regressions fit well, their statistical significance remains low.

### 3.4. Critical Metabolites Associated with Cold Tolerance in Respiratory Metabolism

We sought to identify the metabolites most strongly associated with *S. insularis* larval cold tolerance.

**Figure 4:**
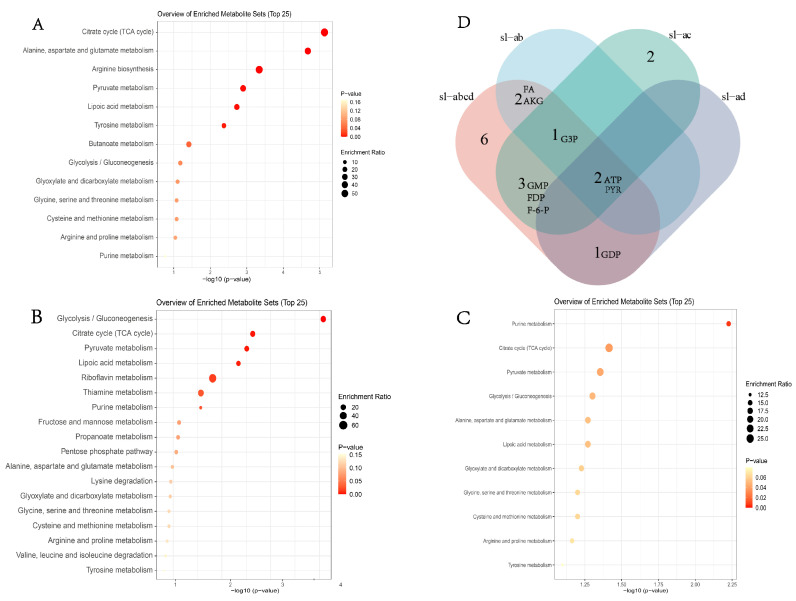
Venn diagram and KEGG pathway enrichment of differential metabolites involved in glycolysis and the TCA cycle. (**D**) Differential metabolites identified in the 0 °C (b), −5 °C (c), and −10 °C (d) treatment groups were compared with the 4 °C (a) control. The Venn diagram shows the overlap and uniqueness of metabolites related to glycolysis and the tricarboxylic acid (TCA) cycle among treatment groups. KEGG enrichment analysis highlights significantly enriched metabolic pathways for differential metabolites between each treatment and the 4 °C control (**A**): 0 °C vs. 4 °C; (**B**): −5 °C vs. 4 °C; (**C**): −10 °C vs. 4 °C.

After excluding metabolic pathways associated with human diseases and drug development, KEGG metabolic pathway enrichment analysis was performed (*p* < 0.05; Figure 4). Significant differential metabolites were primarily enriched in the TCA cycle when comparing the 4 °C and 0 °C groups (Figure 4A). In the comparison between the 4 °C and −5 °C groups, the most pronounced differential metabolites were associated with the glycolytic pathway (Figure 4B). Notably, the comparison between the 4 °C and −10 °C groups revealed that the most significant differential metabolites were related to purine metabolism (Figure 4C).

**Figure 5:**
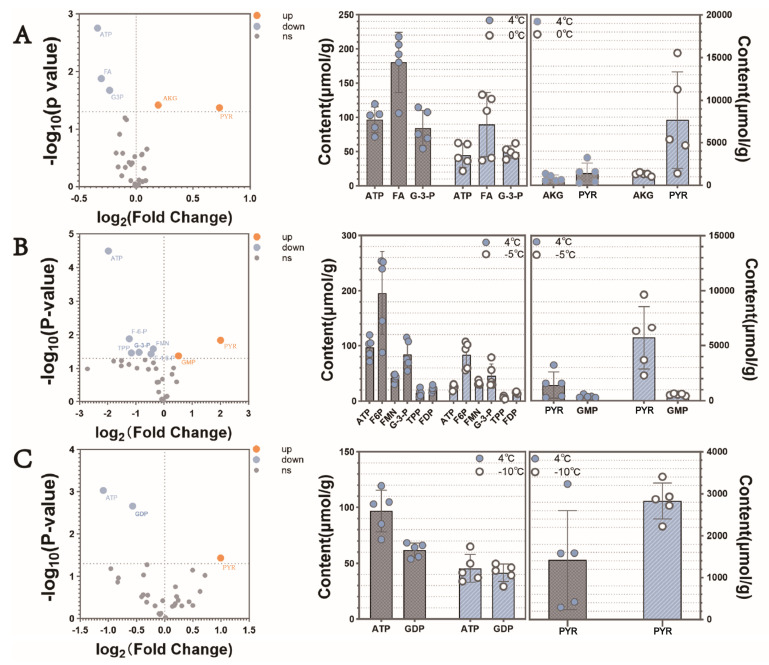
Up- and downregulation of differential metabolites between temperature treatment groups. Differential metabolites were identified across four temperature conditions (4 °C, 0 °C, −5 °C, and −10 °C) based on variable importance in projection (VIP) > 1 and *p* < 0.05. Changes in metabolite abundance were visualized using volcano plots and bar charts to show both the direction (up- or downregulation) and magnitude of regulation. Error bars indicate standard deviations. (**A**): Comparison between 4 °C and 0 °C; (**B**) Comparison between 4 °C and −5 °C. (**C**): Comparison between 4 °C and −10 °C.

A Venn analysis of the ordinary differential metabolites across four temperatures and those identified between the experimental and control groups revealed that ATP and PYR are common significant differential metabolites (Figure 4D). ATP was significantly downregulated, whereas pyruvate was significantly upregulated (Figure 5).

A trend analysis of upregulated and downregulated metabolites was performed to assess intergroup differences between the control group at 4 °C and treatment groups at 0°C, −5 °C, and −10 °C (Figure 5). Significant differences among adenosine triphosphate (ATP) (*p* < 0.05), fenugreek acid (FA) (*p* = 0.013), glycerol 3-phosphate (G-3-P) (*p* = 0.02), α-ketoglutarate (AKG) (*p* = 0.04), and pyruvate (PYR) (*p* = 0.04) were observed when comparing the 4 °C and 0 °C groups (Figure 5A). The comparison between the 4 °C and −5 °C groups revealed significant differences in adenosine triphosphate (ATP) (*p* < 0.05), fructose 6-phosphate (F-6-P) (*p* = 0.014), pyruvate (PYR) (*p* = 0.04), flavin mononucleotide (FMN) (*p* = 0.026), glycerol 3-phosphate (G-3-P) (*p* = 0.033), thiamine pyrophosphate (TPP) (*p* = 0.034), fructose 1,6-diphosphate (FDP) (*p* = 0.037), and guanosine 5-monophosphate (GMP) (*p* = 0.042) (Figure 5B). Adenosine triphosphate (ATP) (*p* < 0.05), guanosine 5-diphosphate (GDP) (*p* < 0.05), and pyruvate (PYR) (*p* = 0.037) exhibited significant differences when comparing the 4 °C and −10 °C groups (Figure 5C).

### 3.5. Transformation Dynamics of Key Metabolites in Response to Low-Temperature Stress

**Figure 6:**
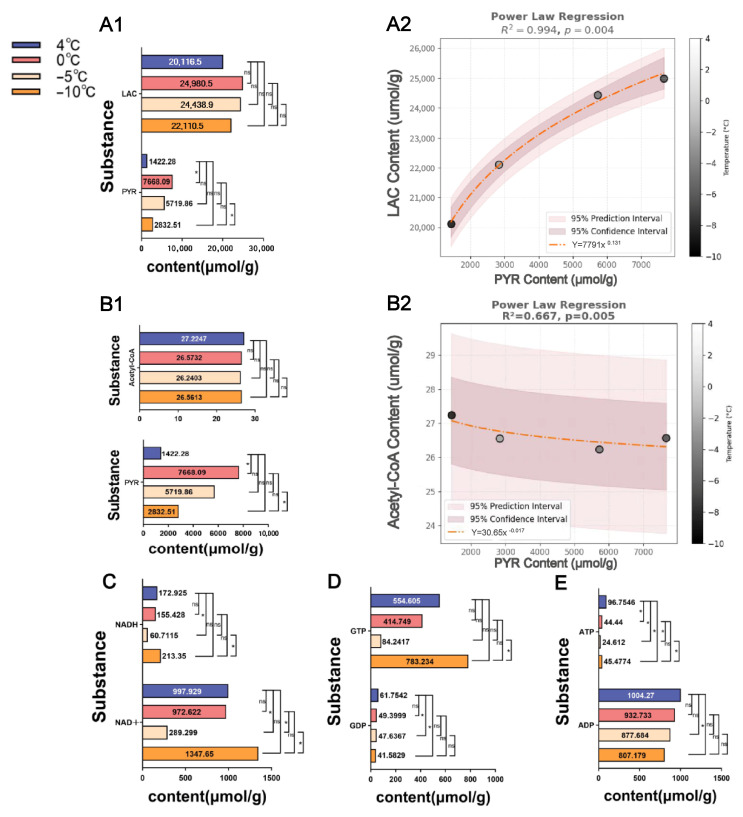
Variations in metabolite content under different temperature conditions. Bar plots (**A**–**E**) display metabolite concentrations at four temperatures (4 °C, 0 °C, −5 °C, and −10 °C), with statistically significant differences indicated (* *p* < 0.05, ns > 0.05) one-way ANOVA followed by LSD post hoc test). Error bars indicate standard deviations. Scatter plots (**A2**,**B2**) illustrate the relationship between temperature and metabolite levels using power-law regression, with 95% confidence and prediction intervals shaded.

Under aerobic conditions, pyruvate (PYR) is converted to acetyl-CoA (Figure 6B1) (F_3,16_ = 0.683, *p* = 0.576), whereas under anaerobic conditions, it is converted to lactate (LAC). The concentrations of both PYR (Figure 6A1,B1) (F_3,16_ = 3.776, *p* < 0.05) and LAC (Figure 6A1) (F_3,16_ = 1.254, *p* = 0.323) peak at 0 °C and reach their lowest levels at 4 °C, with LAC consistently exhibiting higher concentrations than PYR across all temperatures. Acetyl-CoA reaches its maximum concentration at 4 °C and its minimum at −5 °C, maintaining significantly lower concentrations than PYR at all temperature conditions. PYR content exhibits a strong power-law regression with LAC content (Figure 6A2, R^2^ = 0.994, *p* < 0.05), indicating a well-fitted curve where LAC content increases as PYR levels rise. Similarly, PYR and acetyl-CoA content show a significant power-law regression (Figure 6B2, R^2^ = 0.667, *p* < 0.05), with a good fit. However, unlike LAC, acetyl-CoA content decreases gradually as PYR levels increase.

Glycolysis yields two molecules of pyruvate (PYR) and two molecules of ATP, while the tricarboxylic acid (TCA) cycle produces one molecule of GTP. Both metabolic pathways are accompanied by the reduction of NAD^+^ to NADH [20]. In this study, ATP levels (Figure 6E, F_3,16_ = 22.994, *p* < 0.05) were highest at 4 °C, decreased significantly at −5 °C, and then increased markedly at −10 °C. NADH levels (Figure 6C, F_3,16_ = 3.573, *p* < 0.05) significantly declined from 4 °C to −5 °C, followed by a sharp increase, reaching a maximum at −10 °C. GTP levels (Figure 6D, F_3,16_ = 2.884, *p* = 0.068) also decreased from 4 °C to −5 °C and subsequently increased, peaking at −10 °C.

In contrast, ADP levels (Figure 6E, F_3,16_ = 2.187, *p* = 0.129) and NAD^+^ levels (Figure 6C, F_3,16_ = 3.790, *p* < 0.05) consistently remained higher than those of ATP and NADH, respectively. Furthermore, GTP concentrations were significantly lower than GDP levels at all temperatures (Figure 6D, F_3,16_ = 3.494, *p* < 0.05).

### 3.6. Dynamic Changes in Differentially Expressed Genes Under Low-Temperature Stress

**Figure 7:**
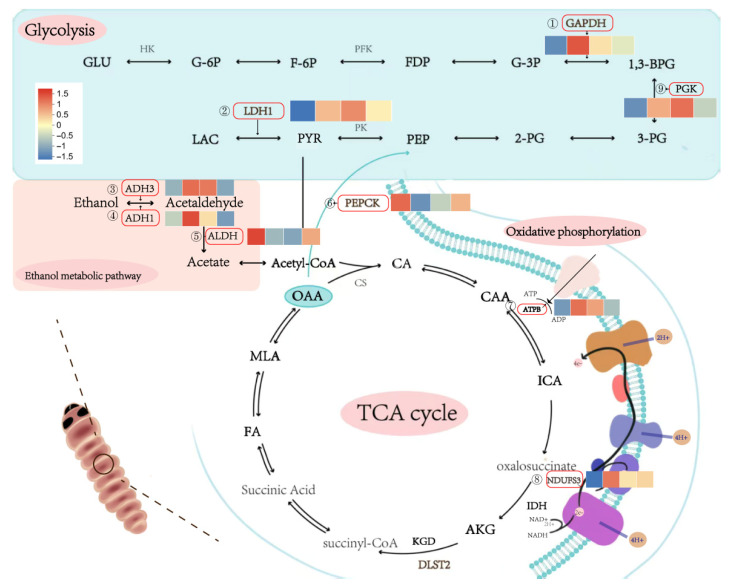
Distribution of key differentially expressed genes in metabolic pathways. The figure includes the tricarboxylic acid (TCA) cycle, glycolysis, oxidative phosphorylation, and ethanol metabolism pathways. A total of nine key differentially expressed metabolic genes were identified, and their functional positions within the pathways are highlighted with red boxes. The expression trends (upregulation or downregulation) under different treatment conditions are visualized using heatmap-style color coding. From left to right, the color blocks represent gene expression under 4 °C, 0 °C, −5 °C, and −10 °C treatments. Abbreviations: *GAPDH*, *glyceraldehyde-3-phosphate dehydrogenase*; *LDH1*, *lactate dehydrogenase 1*; *ADH3*, *alcohol dehydrogenase class 3*; *ADH1, alcohol dehydrogenase 1*; *ALDH*, *aldehyde dehydrogenase*; *PEPCK*, *phosphoenolpyruvate carboxykinase*; *ATPB*, *ATP synthase subunit beta*; *NDUFS3*, *NADH: ubiquinone oxidoreductase core subunit S3*; *PGK*, *phosphoglycerate kinase*.

**Figure 8:**
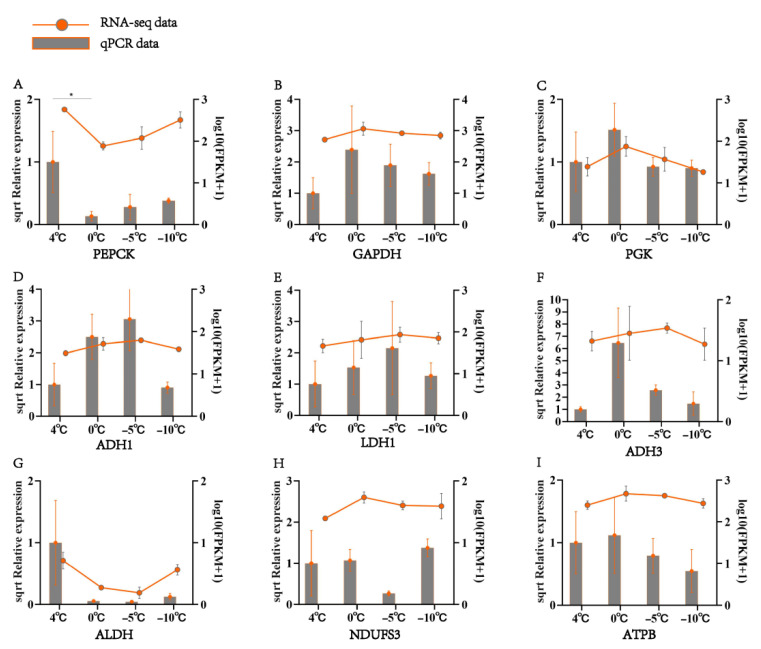
Relative expression levels of selected genes were quantified by quantitative real-time PCR (qRT-PCR) in larvae treated at four temperatures (4 °C, 0 °C, −5 °C, and −10 °C), using the 4 °C group as the control. Expression levels were normalized to the internal reference gene β-actin, and results are presented as normalized expression values. Each bar represents the mean ± standard deviation (SD) of three biological replicates. No statistically significant differences were observed among temperature treatments in the qRT-PCR results for most genes; however, the *PEPCK* gene showed a significant difference between the 4 °C and 0 °C groups (* *p* < 0.05). Overall, the expression trends were generally consistent with the transcriptomic data. Since the selected genes were differentially expressed genes (DEGs) identified from the transcriptome analysis, no additional significance markers were added to the figure to avoid redundancy. (**A**): Trend comparison of *PEPCK* transcriptome and qRT-PCR data. (**B**): Trend comparison of *GAPDH* transcriptome and qRT-PCR data. (**C**): Trend comparison of *PGK* transcriptome and qRT-PCR data. (**D**): Trend comparison of *ADH1* transcriptome and qRT-PCR data. (**E**): Trend comparison of *LDH1* transcriptome and qRT-PCR data. (**F**): Trend comparison of *ADH3* transcriptome and qRT-PCR data. (**G**): Trend comparison of *ALDH* transcriptome and qRT-PCR data. (**H**): Trend comparison of *NDUFS3* transcriptome and qRT-PCR data. (**I**): Trend comparison of *ATPB* transcriptome and qRT-PCR data.

A total of nine differentially expressed genes (DEGs) associated with the tricarboxylic acid (TCA) cycle, glycolysis, pyruvate metabolism, and oxidative phosphorylation pathways were identified from the transcriptome dataset (Figure 7). The qRT-PCR results showed expression patterns largely consistent with the transcriptome data, indicating good concordance (Figure 8).

Most genes showed temperature-dependent expression patterns. *PEPCK* and *ALDH* peaked at 4 °C, declined to their lowest levels at 0 °C and −5 °C, respectively, and then increased again at −10 °C. *GAPDH*, *PGK*, *NDUFS3*, and *ATPB* exhibited a similar trend: expression significantly increased from 4 °C to 0 °C (peaking at 0 °C), followed by a marked or gradual decline to −10 °C. *ADH1*, *ADH3*, and *LDH1* expression levels rose significantly from 4 °C, peaked at −5 °C, and decreased thereafter to their lowest levels at −10 °C.

## 4. Discussion

This study analyzed changes in enzyme activities and related metabolites in *Streltzoviella insularis* larvae under different temperature treatments. Among six rate-limiting enzymes, most exhibited the lowest activity at −5 °C, while hexokinase (HK) and citrate synthase (CS), key enzymes initiating glycolysis and the tricarboxylic acid (TCA) cycle, showed significant responses to cold stress. Pyruvate kinase (PK), isocitrate dehydrogenase (IDH), and α-ketoglutarate dehydrogenase (KGD) displayed a bimodal trend with decreasing temperature, peaking at 0 °C and −10 °C, which may reflect the larvae’s responses to initial cold stress and subsequent cold adaptation. Although no strong correlations were found between rate-limiting enzymes and their upstream or downstream metabolites, pathway-level patterns were observed: glycolytic intermediates (G-6-P, F-6-P, FDP, and PEP) peaked at −10 °C, whereas TCA cycle metabolites (citrate, acetyl-CoA, α-ketoglutarate, and isocitrate) were more abundant at 4 °C or 0 °C. Although the sample size in this study was relatively small, potentially affecting statistical power and the robustness of the results, significant and consistent changes in enzyme activities and metabolites were still observed under these conditions, indicating a clear regulatory effect of temperature on the physiology of *S. insularis* larvae. Moreover, strict experimental design and repeated measurements were employed to maximize data reliability. KEGG enrichment analysis showed significant enrichment of the TCA cycle and glycolysis pathways across different temperature comparisons. Additionally, transcriptomic analysis identified multiple cold-responsive genes involved in these pathways. These findings suggest that key enzymes, metabolites, and regulatory genes may jointly contribute to the cold-tolerance mechanism of *S. insularis* larvae.

### 4.1. Effects of Low-Temperature Stress on Key Enzyme Activities in S. insularis Larval Glycolysis and TCA Cycle

CS and HK are the initial rate-limiting enzymes in the TCA cycle and glycolysis, respectively, and their activities are influenced by temperature and metabolic feedback. Under extreme temperatures, CS activity is generally low in most species [21]. In *Ostrinia nubilalis,* CS activity is highest in non-diapause larvae, suggesting TCA cycle suppression during diapause or cold stress [22]. As a key enzyme at the entry point of the TCA cycle, CS is subject to inhibition by NADH, α-ketoglutarate, and adenine nucleotides (ATP, ADP, AMP) [23]. In this study, CS activity in *S. insularis* larvae decreased significantly with a falling temperature, indicating possible feedback inhibition and a reduced TCA cycle rate under cold conditions. HK, the first enzyme in glycolysis, is similarly regulated. In *Dipetalogaster maximus*, its activity is modulated by temperature through ATP-mediated inhibition [24], while in *Epiblema scudderiana*, cold enhances its affinity for glucose and ATP [25]. G6P, a product of the HK reaction, competitively inhibits substrate binding [26]. In *S. insularis* larvae, HK activity initially increased and then declined with decreasing temperature. Meanwhile, ATP levels first dropped and then rose, and G6P levels increased significantly at −5 °C to −10 °C. These patterns suggest that HK is regulated by metabolite feedback, helping adjust glycolytic flux in response to cold stress.

Researchers have focused on the energy cycle’s response to temperature stress in several studies [5,27,28,29,30]. PK is the final rate-limiting enzyme in the glycolytic pathway. In *Eurosta solidaginis*, PK activity and substrate affinity significantly decrease at low temperatures, leading to a reduction in the metabolic rate [31]. In our study, the PK activity of *S. insularis* larvae increased under the −10 °C treatment, a pattern similar to that observed in certain fish species. For instance, PK levels in *Reinhardtius hippoglossoides* significantly increase during cold stress and freezing conditions [32], while ATP concentration and PK activity in freeze-tolerant *Paralichthys olivaceus* are significantly higher than those in chill-susceptible *P. olivaceus* [33]. The PK activity in *S. insularis* larvae peaks at 0 °C following the onset of cold stress. At this temperature, the substrate PEP reaches its lowest concentration, while the product PYR attains its highest concentration.

IDH is a rate-limiting enzyme in the TCA cycle; however, its role in insect cold tolerance remains largely unexplored. In *Pachycara brachycephalum*, IDH activity at 0 °C is higher than that observed in the control (10 °C) and cold-adapted (5 °C) *Zoarces viviparus* [34]. Regarding *Holothuria scabra* and *H. forskali*, the latter showed a noticeable but non-significant increase in IDH activity after cold treatment, possibly indicating enhanced aerobic metabolism driven by increased energy demand [35]. This finding aligns with our results, where IDH levels in *S. insularis* larvae increased at −10 °C, indicating enhanced metabolic capacity and elevated energy demand. Additionally, IDH may play a role in maintaining organismal homeostasis. NADPH is a key donor for the reducing capacity of reactive oxygen species (ROS) detoxifying enzymes and is produced through various metabolic pathways, including the NADP-dependent IDH reaction. In *Rana sylvatica*, IDH helps sustain NADPH levels during freezing stress, thereby boosting antioxidant defense mechanisms [36]. In *S. insularis* larvae, NADPH levels significantly increased at −10 °C, accompanied by a rise in IDH activity, suggesting that IDH protects against reactive oxygen species in low-temperature environments. KGD regulates the metabolic flux of the TCA cycle and is also sensitive to reactive oxygen species (ROS). The inhibition of this enzyme may lead to metabolic defects resulting from oxidative stress [37]. KGD influences NADH production through its catalytic activity. It catalyzes the conversion of α-ketoglutarate (AKG) to succinyl-CoA, directly producing NADH [37,38]. Thus, the functional state of KGD directly affects NADH production in the TCA cycle, thereby influencing cellular energy metabolism. Both NADH and KGD are essential for intracellular energy metabolism and the generation and clearance of free radicals [39]. NADH contributes to energy production and free radical generation through the electron transport chain, while KGD regulates NADH production via its catalytic reactions, thereby influencing the balance of free radicals. When *S. insularis* larvae were exposed to temperatures below their supercooling point (−10 °C), both NADH levels and KGD activity increased. This suggests that KGD may help maintain homeostasis under low-temperature conditions.

### 4.2. Effects of Low-Temperature Stress on Key Metabolites in S. insularis Larval Glycolysis and TCA Cycle

Metabolomics revealed ATP and pyruvate as shared differential metabolites. Despite limited research on pyruvate in insects, particularly wood-boring pests, and the non-definitive nature of energy metabolites in cold response mechanisms, their dynamic changes merit examination.

GLU is metabolized into PYR and ATP under anaerobic conditions [40]. In cold environments, insects improve their tolerance to hypoxia through metabolic suppression. A reduction in intermediate metabolites or an increase in LAC, signaling a shift to anaerobic metabolism, are characteristics observed in first-instar *Cydia pomonella* larvae under hypoxic conditions [41]. PYR participates in oxidative phosphorylation in the mitochondria and is converted into LAC or alanine as a product of anaerobic glycolysis [31]. As a key product of glycolysis, PYR can undergo oxidative decarboxylation to form acetyl-CoA and fix CO_2_ to generate OAA. In *S. insularis* larvae, PYR content peaked at 0 °C. Across all temperature treatments, LAC levels were consistently higher than PYR, suggesting that anaerobic respiration became the dominant metabolic pathway following cold exposure. Acetyl-CoA levels peaked at 0 °C, but the conversion rate was relatively low compared to total PYR levels. This suggests that while *S. insularis* larvae continued aerobic respiration, their rate was significantly reduced. Similarly, under sublethal low-temperature stress, PYR content increases in *Bactrocera dorsalis* [42]. In our study, PYR levels consistently increased with decreasing temperatures compared to the control group, potentially contributing to enhanced resistance to low-temperature stress. We hypothesize that, following cold stress, PYR is converted into acetyl-CoA and LAC in the larvae, indicating a shift in respiration from predominantly aerobic to a combined aerobic–anaerobic mode. The nonlinear fitting results revealed a strong correlation between PYR, LAC, and acetyl-CoA, likely due to the interrelated and regulated metabolic pathways under specific conditions, such as hypoxia or increased energy demand. The dynamic changes in these metabolites likely reflect cellular adaptation mechanisms to environmental fluctuations, particularly metabolic adjustments in response to hypoxia or cold stress. Studies on *B. dorsalis* suggest that, under sublethal low-temperature stress, insects use neurotransmitters to transition from aerobic to anaerobic metabolism, which reduces TCA cycle activity in the mitochondria and minimizes energy loss [42]. Further research is needed to investigate the role of PYR in insect cold tolerance.

ATP is commonly referred to as the “energy currency” of the cell, as it supplies energy for most biochemical reactions [43]. Overall, ATP levels in *Streltzoviella insularis* larvae remained consistently lower under 0 °C to −10 °C treatments compared to the 4 °C control group. Similarly, in *Drosophila melanogaster*, mitochondrial ATP synthesis rates declined sharply after three days of continuous cold stress [44], suggesting that ATP is extensively consumed during the early stages of cold exposure [45]. However, a significant rebound in ATP levels was observed in larvae under 0 °C to −10 °C conditions. In *Gryllus pennsylvanicus*, prolonged exposure to 0 °C led to a slight increase in muscle ATP levels [46]. In *Penaeus monodon*, ATP concentrations significantly increased following cold stress, indicating a higher energy demand required to withstand low temperatures [47]. This increase may be attributed to ATP production and accumulation via facultative anaerobic glycolysis.

In summary, before reaching the supercooling point, *S. insularis* larvae may lower their metabolic rate to maintain homeostasis, resulting in a decline in ATP levels. However, once temperatures drop below the supercooling point, the cold environment likely triggers an increased energy demand, leading to ATP accumulation for survival. This suggested a significant increase in energy demand in *S. insularis* larvae, indicating a continuous supply of ATP under aerobic and anaerobic conditions.

The trends in ATP and GTP levels followed a similar pattern: total ATP content was lower than ADP, whereas total GTP content exceeded GDP. GTP can be converted into ATP, serving as an additional energy source for the organism. However, under cold conditions, increased energy demand leads to higher ATP consumption compared to GTP. In *S. insularis* larvae, NAD+ and NADH followed similar trends, with NAD+ levels significantly higher than those of NADH. Both metabolites peaked at −10 °C. The sustained availability of NADH suggests a sufficient substrate supply, helping to maintain homeostasis in the organism. However, there was limited research on the functions of these two metabolites in insects subjected to low temperatures. Conversely, studies on marine ectotherms, including bony fish, scallops, and mussels, suggest that sirtuins (NAD+-dependent deacetylases) act as energy sensors, becoming active under caloric restriction. Sirtuins target and regulate cellular metabolism, diminish oxidative stress, and modulate cellular stress responses [48].

### 4.3. Complexity of Enzyme–Substrate Relationships in Glycolysis and the TCA Cycle

In glycolytic pathways, the relationship between enzyme activity and substrate concentration may be described using a quadratic polynomial regression model. Although significant correlations (high R^2^ values) have been observed between phosphofructokinase (PFK) and pyruvate kinase (PK) and their respective substrates and products, the relatively high *p*-values suggest that this relationship is influenced by additional unmeasured factors, such as temperature, oxygen concentration, pH, and nutritional status [49,50]. Many biological systems exhibit regulatory mechanisms, including negative feedback inhibition and compensatory metabolic pathways, which may further complicate direct enzyme–substrate interactions [51,52]. The relationship between enzymes and substrates in the TCA cycle cannot be explained by a single quadratic polynomial model, possibly due to its dependence on oxygen concentration. As the core of aerobic metabolism, the TCA cycle is influenced by oxygen availability, mitochondrial function, and metabolic remodeling under cold conditions. Low temperatures reduce mitochondrial respiration rates, decreasing TCA cycle flux [53,54]. They may also induce partial anaerobic metabolism, altering substrate availability. Additionally, reduced oxidative phosphorylation efficiency regulates TCA cycle enzymes through energy-sensing mechanisms, further complicating enzyme–substrate interactions [55]. These factors together prevent the TCA cycle from being accurately described by a single linear or nonlinear regression model.

### 4.4. Role of Differentially Expressed Metabolic Genes in S. insularis Larval Cold Tolerance

ATP and PYR were identified as core differential metabolites under cold stress, with their associated genes predominantly enriched in the pyruvate metabolism and oxidative phosphorylation pathways. Several candidate genes were annotated in both pyruvate and ethanol metabolism, the latter being a downstream branch of the former. The observed expression patterns under low-temperature conditions suggest a metabolic shift toward anaerobic energy production.

*PEPCK*, the gene encoding a key enzyme that catalyzes the conversion of OAA to PEP, was significantly upregulated from 0 °C to −10 °C. This gene has been previously reported to be cold-responsive in *Belgica antarctica* [56], and it is also associated with gluconeogenesis and antifreeze compound synthesis in *Eocanthecona furcellata* [57], indicating that it may perform a similar function in this study. Notably, *PEPCK* was differentially expressed across four essential pathways—namely, the TCA cycle, glycolysis, pyruvate metabolism, and oxidative phosphorylation—highlighting its coordinated regulatory role in cold adaptation.

In combination with the expression trends of *LDH1* and *ATPB*, these results support a respiratory shift from complete aerobic metabolism to a mixed aerobic–anaerobic mode during overwintering. *LDH1* was significantly upregulated at low temperatures, indicating enhanced conversion of pyruvate to lactate. This change is consistent with the increased LDH activity observed during diapause and cold acclimation in *Dendroctonus armandi* [58], which contributes to anaerobic energy metabolism under cold conditions. Therefore, the upregulation of *LDH1* is likely a crucial strategy for cold adaptation in insects. Interestingly, *ATPB*, which encodes the β subunit of ATP synthase and is directly involved in ATP production, showed an expression pattern opposite to ATP content. In *Hyles euphorbiae*, cold adaptation involves the downregulation of ATP synthase genes as part of global metabolic suppression [59]. Similarly, in *Drosophila melanogaster*, cold stress reduces mitochondrial ATP production and survival in non-acclimated individuals, whereas cold-acclimated flies maintain higher mitochondrial coupling efficiency and ATP synthesis rates [60].

It is proposed that at 0 °C, the upregulation of *ATPB* may represent a compensatory response to cold stress. However, due to metabolic suppression and impaired mitochondrial function, ATP levels still declined. As the temperature dropped further, *ATPB* expression decreased while ATP content increased, possibly due to reduced ATP consumption or compensation via enhanced glycolysis. This pattern reflects the complex regulatory mechanisms of ATP homeostasis under cold conditions.

### 4.5. Conclusions and Perspectives

In conclusion, our study demonstrates that *S. insularis* larvae maintain energy homeostasis under cold stress by flexibly switching between aerobic and anaerobic metabolic pathways, providing new insights into insect overwintering mechanisms from a metabolic perspective. The expression patterns of *ATPB*, *PEPCK*, and *LDH1* suggest that these genes may play important roles in cold tolerance. Future research could further investigate the dynamic expression changes in these genes during the overwintering process and employ molecular tools such as gene editing or transcriptional interference to elucidate their physiological functions. Additionally, comparative studies across different regions or species may reveal common or divergent cold response mechanisms in insects.

Although further experimental validation is needed, targeting these key genes involved in overwintering metabolism may offer new targets and strategies for pest control.

## Figures and Tables

**Table 1:** Corresponding abbreviations of rate-limiting enzymes.

Rate-Limiting Enzyme Names	Acronyms
hexokinase	HK
phosphofructokinase	PFK
pyruvate kinase	PK
citrate synthase	CS
isocitrate dehydrogenase	IDH
α-ketoglutarate dehydrogenase	KGD

**Table 2:** Corresponding abbreviations of metabolites.

Metabolic Pathway	Metabolites	Acronyms
Glycolytic	glucose	GLU
Glycolytic	glucose-6-phosphate	G-6-P
Glycolytic	fructose-6-phosphate	F-6-P
Glycolytic	1,6-fructose-diphosphate	FDP
Glycolytic	phosphoenolpyruvic acid	PEP
Glycolytic	pyruvic acid	PYR
Glycolytic	lactic acid	LAC
Glycolytic	adenosine triphosphate	ATP
Glycolytic	adenosine diphosphate	ADP
TCA cycle	acetoacetyl-CoA	Acetyl-CoA
TCA cycle	oxaloacetic acid	OAA
TCA cycle	citric acid	CA
TCA cycle	isocitric acid	ICA
TCA cycle	α-ketoglutaric acid	AKG
TCA cycle	guanosine triphosphate	GTP
TCA cycle	guanosine diphosphate	GDP
TCA cycle	nicotinamide adenine dinucleotide (reduced)	NADH
TCA cycle	nicotinamide adenine dinucleotide (oxidized)	NAD+

## Data Availability

Data for “Metabolic Remodeling of the Tricarboxylic Acid Cycle and Glycolysis Reveals Cold-Induced Respiratory Adaptations in *Streltzoviella insularis* (Staudinger) (Lepidoptera: Cossidae) Larvae” is available in Mendeley Data. (DOI: https://doi.org/10.17632/j543hyxkk4.1) (https://data.mendeley.com/drafts/j543hyxkk4, accessed on 12 November 2024).

## Supplementary Materials

The following supporting information can be downloaded at: https://www.mdpi.com/article/10.3390/insects16080864/s1, Table S1. List of all additives in the laboratory *S. insularis* larvae feed. Table S2. Abbreviations of corresponding differentially expressed genes.
