# Supplementary material for: Metabolic Remodeling of the Tricarboxylic Acid Cycle and Glycolysis Reveals Cold-Induced Respiratory Adaptations in Streltzoviella insularis (Staudinger) (Lepidoptera: Cossidae) Larvae
Source: Insects. 2025 Aug 19;16(8):864. doi: 10.3390/insects16080864 (PMC12386693; doi:10.3390/insects16080864)
Supplement: Supplementary file 1 [file insects-16-00864-s001.zip › insects-3786333-supplementary.pdf]

Table S1. List of all additives in the laboratory *S. insularis* larvae feed.

Additive	Dosage	Origin
water	70ml	
F. pennsylvanica wood flour	30g	
corn flour	30g	
soybean flour	50g	
sucrose	20g	Beekman Biotechnology Co., Ltd., Hunan, China
glucose	20g	Beekman Biotechnology Co., Ltd., Hunan, China
agar powder	6g	Marel Biochemical Science and Technology Co., Ltd., Shanghai, China
Vesuvius salt	4g	Bonderrey Biotechnology Co., Ltd., Beijing, China
cholesterol	1g	Solepol Science and Technology Co., Ltd., Beijing
potassium sorbate	1g	Myriad Biochemical Technology Co., Ltd., Shanghai
methyl p-hydroxybenzoate	0.9g	Yuan Ye Biotechnology Co., Ltd., Beijing
vitamin mix	0.4g	Wyeth Pharmaceuticals Co., Ltd., Suzhou

Table S2. Abbreviations of corresponding differentially expressed genes.

GAPDH	Glyceraldehyde-3-phosphate dehydrogenase
LDH1	Lactate dehydrogenase 1
ADH3	Alcohol dehydrogenase class 3
ADH1	Alcohol dehydrogenase 1
ALDH	Aldehyde dehydrogenase
PEPCK	Phosphoenolpyruvate carboxykinase
ATPB	ATP synthase subunit beta
NDUFS3	NADH:ubiquinone oxidoreductase core subunit S3
PGK	Phosphoglycerate kinase

1. CS activity

Citrate synthase (CS) activity was determined using a commercial ELISA kit (YT-927855O2/48T, Jiangsu Yutong Biotechnology Co., Ltd.) based on a double-antibody sandwich method. Standard solutions were prepared by serial dilution as described in the table below.

Three types of wells were included: blank (no sample or enzyme reagent), standard, and sample. For each standard well, 50 μL of diluted standard was added. Sample wells received 40 μL of diluent and 10 μL of test sample (final 5× dilution). Samples were dispensed carefully at the bottom of each well and gently mixed.

Plates were sealed and incubated at 37°C for 30 minutes, then washed five times with 30× diluted wash buffer. After drying, 50 μL of enzyme conjugate was added (excluding blank wells), followed by repeated incubation and washing.

Next, 50 μL each of chromogenic reagents A and B was added, mixed, and incubated at 37°C in the dark for 10 minutes. Finally, 50 μL of stop solution was added to terminate the reaction, changing the color from blue to yellow. Absorbance was measured at 450 nm within 15 minutes, using the blank as a reference.

CS concentration was calculated from a standard curve plotted with OD values versus standard concentrations, or using the regression equation. Final concentrations were adjusted by the dilution factor.

24IU/L	Standard Solution No. 5	In total, 150 μL of undiluted standard solution was mixed with 150 μL of standard diluent.
12IU/L	Standard Solution No. 4	In total, 150 μL of Standard Solution No. 5 was mixed with 150 μL of standard diluent.
6IU/L	Standard Solution No. 3	In total, 150 μL of Standard Solution No. 4 was mixed with 150 μL of standard diluent.
3IU/L	Standard Solution No. 2	In total, 150 μL of Standard Solution No. 3 was mixed with 150 μL of standard diluent.
1.5IU/L	Standard Solution No. 1	In total, 150 μL of Standard Solution No. 2 was mixed with 150 μL of standard diluent.

2. HK activity

The activity of hexokinase (HK) was measured using a commercial enzyme-linked immunosorbent assay (ELISA) kit specifically for HK, based on a double-antibody sandwich method (HK ELISA Kit, [manufacturer details if available]). This kit quantitatively detects the level of HK in biological samples.

Standard solutions were prepared by serial dilution as described in the table below. The specific experimental procedures and calculation methods for HK activity were identical to those described for citrate synthase (CS) activity measurement.

80IU/L	Standard Solution No. 5	In total, 150 μL of undiluted standard solution was mixed with 150 μL of standard diluent.
40IU/L	Standard Solution No. 4	In total, 150 μL of Standard Solution No. 5 was mixed with 150 μL of standard diluent.
20IU/L	Standard Solution No. 3	In total, 150 μL of Standard Solution No. 4 was mixed with 150 μL of standard diluent.
10IU/L	Standard Solution No. 2	In total, 150 μL of Standard Solution No. 3 was mixed with 150 μL of standard diluent.
5IU/L	Standard Solution No. 1	In total, 150 μL of Standard Solution No. 2 was mixed with 150 μL of standard diluent.

3. KGD activity

KGD activity was measured using a commercial kit (BC0710-50T/48S, Beijing Solarbio Technology Co., Ltd.). A 0.1 g larval tissue sample was homogenized on ice with 1 mL of Reagent I and 10 μL of Reagent II, then centrifuged at 4° C, 11,000 rcf for 10 min. The supernatant was collected and kept on ice. A microplate reader was preheated for 30 min. For the blank, 200 μL of working solution was incubated at 37°C for 5 min, followed by the addition of 8 μL of Reagent VIII and 12 μL of distilled water. Absorbance at 340 nm was recorded at 10 s (A1) and 2 min 10 s (A2), and ΔA_blank = A2 − A1.

For the sample, the supernatant replaced water, and absorbance was measured at 10 s (A3) and 2 min 10 s (A4), with $\Delta A\_sample = A4 - A3$. KGD activity was then calculated.

KGD activity (U/g) = 1488.5 × ($\Delta A\_sample - \Delta A\_blank$) / W, where W is the sample weight (g).

4. PFK activity

PFK activity was measured using a commercial assay kit (BC0530-50T/48S, Beijing Solarbio Technology Co., Ltd.). A 0.1 g sample of larval tissue was weighed, mixed with 1 mL of extraction buffer (Phosphofructokinase Activity Assay Kit), and homogenized on ice. The homogenate was then centrifuged at 4°C at 8,000 rcf for 10 minutes, and the supernatant was collected and kept on ice. The microplate reader was preheated for 30 minutes prior to the assay. For the assay, 10 μL of the sample, 10 μL of Reagent III, 10 μL of Reagent IV, and 170 μL of Reagent II (Phosphofructokinase Activity Assay Kit) were added to a 96-well plate. The absorbance at 340 nm was measured at 20 seconds (A1) and again after a 10-minute reaction (A2). The change in absorbance (ΔA) was calculated as $\Delta A = A1 - A2$.

The phosphofructokinase (PFK) activity was calculated using the following formula: PFK activity (U/g tissue) = 321 × ΔA / W, where W represents the sample weight (g).

5. PK activity

Pyruvate kinase (PK) activity was measured using a commercial assay kit (AC10162-50T/48S, Shanghai Jizi Biochemical Technology Co., Ltd.). A 0.1 g sample of larval tissue was homogenized on ice in 1 mL of extraction buffer (Pyruvate Kinase Activity Assay Kit) and then centrifuged at 4°C at 8,000 rcf for 10 minutes. The resulting supernatant was collected and kept on ice. For the assay, 180 μL of working solution, 6 μL of Reagent III, 3 μL of Reagent IV (Pyruvate Kinase Activity Assay Kit), and 6 μL of the sample were added to a 96-well plate. Absorbance at 340 nm was measured at 20 seconds (A1) and again after 2 minutes and 20 seconds (A2). The absorbance change (ΔA) was calculated as $\Delta A = A1 - A2$.

The pyruvate kinase (PK) activity was determined using the following formula:

PK activity (U/g tissue) = 2613 × ΔA / W, where W represents the sample weight (g).

6. IDH activity

IDH activity was measured using a commercial assay kit (AK328-50T/48S, Beijing Boosun Biotechnology Co., Ltd.). A 0.1 g tissue sample was homogenized on ice in 1 mL of AK328-A and 10 μL of AK328-C. The homogenate was centrifuged at 4°C, 600 rcf for 5 minutes, and the supernatant was collected and further centrifuged at 11,000 rcf for 10 minutes at 4°C.

The resulting pellet was resuspended in 200 μL of AK328-B and 2 μL of AK328-C, then disrupted by ultrasonication in an ice bath (200 W, 3 s on, 10 s off, 30 cycles).

The working solution was pre-incubated at 25°C for 5 minutes. Then, 12 μL of AK328-G, 16 μL of the sample, and 200 μL of the working solution were added to a 96-well plate. Absorbance at 340 nm was recorded at 20 seconds (A1) and again at 2 minutes and 20 seconds (A2). The change in absorbance was calculated as $\Delta A = A1 - A2$.

IDH activity was calculated using the following formula:

ICDHm activity (nmol/min/g fresh weight) = 231.3 × ΔA / W, where ΔA is the change in absorbance and W is the sample weight in grams.

7. Determination of Glucose Content

Glucose content was measured using a commercial kit (R30282-100T, Shanghai Yuanye Biotechnology Co., Ltd.). Approximately 0.1 g of tissue was homogenized in 0.9 mL of PBS on

ice, centrifuged at 2500–3000 rcf for 10 min, and the supernatant was collected. Reaction mixtures were prepared as follows:

Blank: 10 μL of 5% trichloroacetic acid + 500 μL O-Toluidine reagent.

Standard: 9 μL of 5% trichloroacetic acid + 1 μL glucose standard + 500 μL O-Toluidine reagent.

Sample: 10 μL of supernatant + 500 μL O-Toluidine reagent.

After mixing, tubes were boiled for 15 min, cooled rapidly in ice water, and 255 μL of each reaction was transferred to a 96-well plate. Absorbance was measured at 630 nm, and glucose (GLU) content was calculated accordingly.

Astandard/Ameasured×5, where A_standard is the absorbance of the standard solution, and A_measured is the absorbance of the test sample.
